# Noncanonical feedback loop between “RIP3–MLKL” and “4EBP1–eIF4E” promotes neuronal necroptosis

**DOI:** 10.1002/mco2.70107

**Published:** 2025-02-18

**Authors:** Shuchao Wang, Yun Zhang, Meijuan Wang, Zhihao Zhai, Yating Tan, Weiye Xu, Xiaozhen Ren, Ximin Hu, Jinyou Mo, Jia Liu, Yunfeng Yang, Dan Chen, Bing Jiang, Hualin Huang, Jufang Huang, Kun Xiong

**Affiliations:** ^1^ Department of Ophthalmology The Second Xiangya Hospital of Central South University Changsha Hunan China; ^2^ Center for Medical Research The Second Xiangya Hospital of Central South University Changsha Hunan China; ^3^ National Clinical Research Center for Mental Disorders The Second Xiangya Hospital of Central South University Changsha Hunan China; ^4^ National Center for Mental Disorders The Second Xiangya Hospital of Central South University Changsha Hunan China; ^5^ Department of Anesthesiology The Second Xiangya Hospital of Central South University Changsha Hunan China; ^6^ Department of Anatomy and Neurobiology, Xiangya School of Basic Medical Sciences Central South University Changsha Hunan China; ^7^ Medical Imaging Center Qingdao West Coast New District People's Hospital Qingdao Shandong China; ^8^ Department of Neurosurgery The Eighth Affiliated Hospital Sun Yat‐Sen University Shenzhen China; ^9^ Hunan Key Laboratory of Ophthalmology Changsha Hunan China; ^10^ Hunan Clinical Research Center of Ophthalmic Disease Changsha Hunan China; ^11^ Reproductive Medicine Center, Department of Obstetrics and Gynecology The Second Xiangya Hospital of Central South University Changsha Hunan China; ^12^ Department of Radiology The Second Xiangya Hospital of Central South University Changsha Hunan China; ^13^ Biobank of the Second Xiangya Hospital of Central South University Changsha Hunan China

**Keywords:** 4EBP1, eIF4E, MLKL, necroptosis, RIP3, stroke

## Abstract

Stroke is a leading risk factor for disability and death. Necroptosis is involved in stroke pathogenesis. However, the molecular mechanisms underlying necroptosis in stroke remain unclear. The mammalian target of rapamycin complex 1 (mTORC1) modulates necroptosis in the gut epithelium. Eukaryotic translation initiation factor 4E (eIF4E)‐binding protein‐1 (4EPB1) is one of the main downstream molecules of mTORC1. This study addresses the role of the 4EBP1–eIF4E pathway in necroptosis. The 4EBP1–eIF4E pathway was found to be activated in both necroptotic HT‐22 and mouse middle cerebral artery occlusion (MCAO) models. Functionally, 4EBP1 overexpression, eIF4E knockdown, and eIF4E inhibition suppressed necroptosis, respectively. Furthermore, a positive feedback circuit was observed between the 4EBP1–eIF4E and receptor‐interacting protein‐3 (RIP3)–mixed lineage kinase domain‐like protein (MLKL) pathways, in which RIP3–MLKL activates the 4EBP1–eIF4E pathway by degrading 4EBP1 and activating eIF4E. This in turn enhanced RIP3–MLKL pathway activation. The eIF4E activation derived from this loop may stimulate cytokine production, which is a key factor associated with necroptosis. Finally, using a mouse MCAO model, the application of eIF4E, RIP3, and MLKL inhibitors was found to have a regulatory mechanism similar to that in the in vitro study, reducing the infarct volume and improving neurological function in MCAO mice.

## Graphical abstract

Schematic depicting the canonical and noncanonical mammalian target of rapamycin (mTOR) and necroptotic pathway. (A) Canonical mTOR pathway. (B) Noncanonical and positive amplification loop between “RIP3–MLKL” and “4EBP1–eIF4E” pathways in necroptosis. (C) Canonical RIP3–MLKL‐mediated necroptosis.

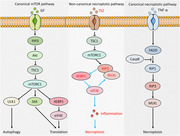

## 1 INTRODUCTION

Stroke is a leading risk factor for disability and death when effective treatment is not.^1^, ^2^, ^3^ However, the pathogenesis of neuronal damage in the brain following stroke is highly complex.^1^, ^2^ Reduced blood flow caused by stroke immediately leads to energy deficiency, ion imbalances, and metabolic disorders, resulting in a series of detrimental outcomes, including excitotoxicity, inflammation, and oxidative stress, which can lead to the death of neurons and non‐neuronal cells in the brain, ultimately resulting in motor dysfunction and cognitive impairment, among others. Middle cerebral artery occlusion (MCAO) is widely used to investigate the cellular regulatory mechanisms of neuronal death in stroke and to provide neuroprotection against stroke.^5^ Recently, significant progress has been made in understanding several molecular targets for pharmacological neuroprotection.^1^ However, the treatment effect is much lower than expected in certain subsets of stroke patients.^1^, ^2^

Necroptosis is a type of regulated cell necrosis that is induced by tumor necrosis factor (TNF) receptor activation, mediated by receptor‐interacting protein‐1 (RIP1), RIP3, and mixed lineage kinase domain‐like protein (MLKL), independent of caspases, and morphologically similar to necrosis, such as organelle swelling, disruption of cytomembrane integrity, and leakage of intracellular contents.^6^, ^7^, ^8^, ^9^ In various disorders, such as hypoxia and ischemia, necroptosis can be triggered by extracellular stimuli that converge on RIP3 and RIP1 necrosome. Subsequently, activated RIP3 recruits and phosphorylates its downstream target, MLKL, which translocates to the cytomembrane, leading to necrosis.^6^, ^7^, ^8^ The traditional and classical inductive method for necroptosis is the combined application of TNF‐α, Smac mimics, and Z‐VAD‐FMK (TSZ).^7^, ^8^, ^10^ In the necroptotic process, caspase‐8 inhibition by Z‐VAD‐FMK is necessary to induce necroptosis.^7^, ^10^ Although previous studies have indicated a key role of the RIP3–MLKL pathway in necroptosis, the upstream and downstream pathways in necroptotic pathogenesis remain unclear, necessitating further studies. Necroptosis is involved in the pathogenesis of neuronal death after various injuries to the brain.^11^, ^12^, ^13^, ^14^, ^15^ Pharmacological analysis has revealed that treatment with Necrostatin‐1 (Nec‐1), a necroptosis inhibitor, can reduce neuronal necroptosis and infarct volume in MCAO.^8^, ^11^, ^16^ However, studies on the molecular mechanisms that play vital roles in the necroptotic pathway in neuropathological processes remain scarce.

The mammalian target of rapamycin (mTOR) is a serine/threonine protein kinase that converges a series of extracellular and intracellular signals, including glucose, amino acids, cellular stress, and cytokines, to modulate basic physiological activities in mammals, such as playing vital functions in cell proliferation, growth, development.^17^ By binding to other proteins, mTOR forms two main complexes: mTOR complex 1 (mTORC1), comprising RAPTOR, mLST8, PRAS40, and DEPTOR, and mTORC2, comprising mLST8, mSin1, DEPTOR, Protor, and Rictor.^17^ mTOR signaling is considered an important regulator in many contexts because of its role in sensing and converging signals from the surrounding microenvironment.^17^, ^18^ Emerging evidence has shown that mTORC1 participates in necroptosis.^18^, ^19^, ^20^ For example, ablation of an amino acid surplus or tuberous sclerosis 1 (TSC1) leads to mTORC1 activation in intestinal epithelial cells (IECs), which results in aberrant RIP3 and MLKL activation, increased necroptosis, and homeostasis dysfunction in the gut epithelium, frequently caused by a western diet, intestinal dysbiosis, and genetic alterations.^19^ mTORC1 activation is primarily reflected by increased phosphorylation of three major substrates: eukaryotic translation initiation factor 4E (eIF4E)‐binding protein‐1 (4EPB1), p70 ribosomal S6 kinase (S6K), and Unc‐51‐like kinase 1 (ULK1).^17^, ^21^ The canonical function of mTORC1 is to regulate protein synthesis via 4EBP1 and S6K phosphorylation during cell proliferation and growth.^17^, ^22^ Specifically, mTORC1 promotes cap‐dependent translation through phosphorylating and inhibiting 4EBP1, leading to dissociating from eIF4E to initiate translation.^17^ Under sufficient nutrient conditions, mTORC1 inhibits autophagy by phosphorylating and inactivating ULK1.^17^, ^21^ Previous evidence has also indicated that ULK1 promotes RIP1 phosphorylation at Ser357 and autophagy, leading to necroptosis inhibition.^23^ ULK1 overexpression can also lead to reduced necroptosis in Wilson's disease.^24^ According to reports that mTORC1 and ULK1 play key roles in the pathogenesis of necroptosis, there is no direct evidence of a connection between necroptosis and 4EBP1/S6K, two other key substrates of mTORC1.

Recent studies have shown that p90 ribosomal S6 kinase 1 (RSK1) plays a vital role in necroptosis.^10^ RSK1 can be activated in TNF‐treated cells via the pyruvate dehydrogenase kinase 1 (PDK1)‐dependent mechanism.^21^ Activated RSK1 is then recruited to stabilize necrosome, triggering necroptosis.^11^ Furthermore, the inhibition of both PDK1 and RSK1 was found to significantly attenuate necroptosis in TNF‐treated L929 cells, protecting mice from TNF‐induced cecum injury.^10^ Consistent with this report, our previous studies have found that RSK3 plays a regulatory role in RIP3‐ and MLKL‐modulated neuronal necroptosis in a glaucoma model.^25^ After the suppression of both RSK3 activity and expression, the number of necroptotic neurons was reduced in rat retinal neurons.^25^ Notably, both RSK and S6K belong to the AGC kinase superfamily and have similar biological functions, such as coordinating protein synthesis coordinately.^26^ Combined with reports that PDK1 is a known kinase that can be activated by S6K,^26^, ^27^ S6K and 4EBP1, two other mTOR substrates, were speculated to also play an important role in necroptosis; however, this requires further study.

This study focused on addressing the role of 4EBP1–eIF4E signaling in necroptosis and discussed the role of S6K in another study. The 4EBP1–eIF4E pathway was found to be activated in both necroptotic HT‐22 and mouse MCAO models. Functionally, 4EBP1 overexpression, eIF4E knockdown, and pharmacological inhibition restrained the progression of necroptosis induced by TSZ. Furthermore, a positive feedback circuit between the 4EBP1–eIF4E and RIP3–MLKL pathways was revealed, in which RIP3–MLKL activates the 4EBP1–eIF4E pathway by degrading 4EBP1 and activating eIF4E, which in turn enhances RIP3–MLKL pathway activation. The activation of eIF4E derived from this loop may stimulate cytokine production, which is a key factor associated with necroptosis. Finally, in a mouse MCAO model study, the application of eIF4E, RIP3, and MLKL inhibitors was found to exhibit a regulatory mechanism similar to that observed in the in vitro study. Collectively, these findings suggest a noncanonical and positive amplification loop between the “RIP3–MLKL” and “4EBP1–eIF4E” pathways in the process of necroptosis and reveal a potential therapeutic target for necroptosis treatment in central nervous system (CNS) injuries or other diseases.

## 2 RESULTS

### 2.1 TSZ induces necroptosis in HT22 neuronal cells

**FIGURE 1:**
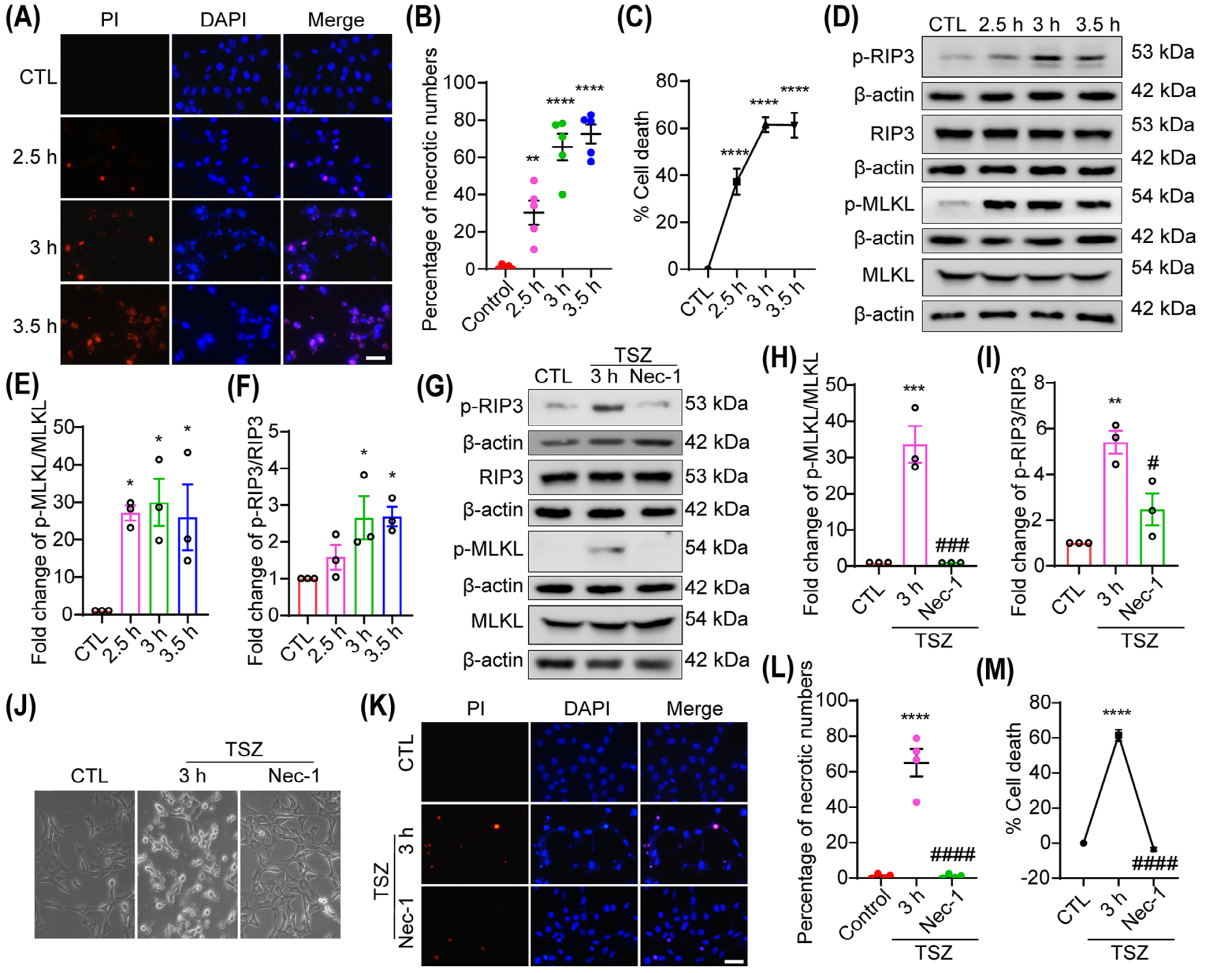
TNF‐α, Smac mimics, and Z‐VAD‐FMK (TSZ) induces necroptosis in HT22 neuronal cells. (A, K) TSZ or Nec‐1 pretreated HT22 cells were stained with propidium iodide (PI; red) after TSZ treatment. Nuclei were labeled with DAPI (blue). (B, L) Number of PI‐positive necrotic cells. (C, M) The percentage of necrotic cells was determined by lactate dehydrogenase (LDH) assay, *n* = 4. (D, G) Western blot showing the levels of p‐receptor‐interacting protein‐3 (RIP3)/RIP3 and p‐mixed lineage kinase domain‐like protein (MLKL)/MLKL expression in HT22 cells following TSZ treatment or pretreatment with Nec‐1 before TSZ treatment. (E, F, H, I) Statistical analysis results of p‐RIP3/RIP3 and p‐MLKL/MLKL expression. (J) Phase‐contrast images showing the morphological changes in necroptotic cells treated with TSZ or Nec‐1. Data are expressed as the mean ± SD (*n* = 3, 4, or 5). *p < 0.05, **p < 0.01, ***p < 0.005, ****p < 0.001 versus control (CTL) group. *#p *< 0.05, *###p *< 0.005, *####p* < 0.001 versus TSZ 3 h group. Two‐tailed unpaired Student's *t*‐test (H, I, L, M). Scale bar: 50 µm in all panels.

First, whether TSZ induces necroptosis in HT22 cells was examined. HT22 cells were treated with TSZ for 2.5, 3, or 3.5 h. The propidium iodide (PI) staining results showed that the number of PI‐positive necrotic cells increased at the observed time points (Figure 1A,B). A lactate dehydrogenase (LDH) release assay was performed to evaluate the percentage of necrotic HT22 cells, indicating that necrosis significantly increased at the observed time points (Figure 1C). Activation of RIP3 and MLKL are key markers of necroptosis. Western blot analysis showed that p‐RIP3 and p‐MLKL levels increased and peaked at 3 h following TSZ treatment, indicating the activation of RIP3 and MLKL during TSZ‐induced HT22 cell necrosis (Figure 1D–F). In subsequent experiments, 3 h was chosen as the primary intervention time point. Nec‐1 is a specific necroptosis inhibitor. Therefore, the effects of Nec‐1 on TSZ‐induced necrosis in HT22 cells were evaluated. The western blotting results showed that RIP3 and MLKL activation was inhibited following Nec‐1 treatment (Figure 1G–I), indicating that necroptosis occurred in TSZ‐induced HT22 cells. Phase‐contrast images further showed that TSZ triggered an increase in necroptotic cells, characterized by cell swelling and rupture, which was inhibited by Nec‐1 (Figure 1J). In addition, the PI staining and LDH assay results showed that necrotic HT22 cells were reduced after Nec‐1 treatment (Figure 1K–M). To further verify that RIP3 and MLKL activation is specific to necroptosis, HT22 cells were exposed to TS, an apoptosis inducer, to exclude the possibility that RIP3 and MLKL were activated during the apoptotic process. The results showed that the expression and phosphorylation levels of RIP3 and MLKL were not significantly altered in apoptotic HT22 cells (Figure S1A–C), further indicating that TSZ specifically induced necroptosis.

### 2.2 eIF4E knockdown and inhibition decrease necroptosis

**FIGURE 2:**
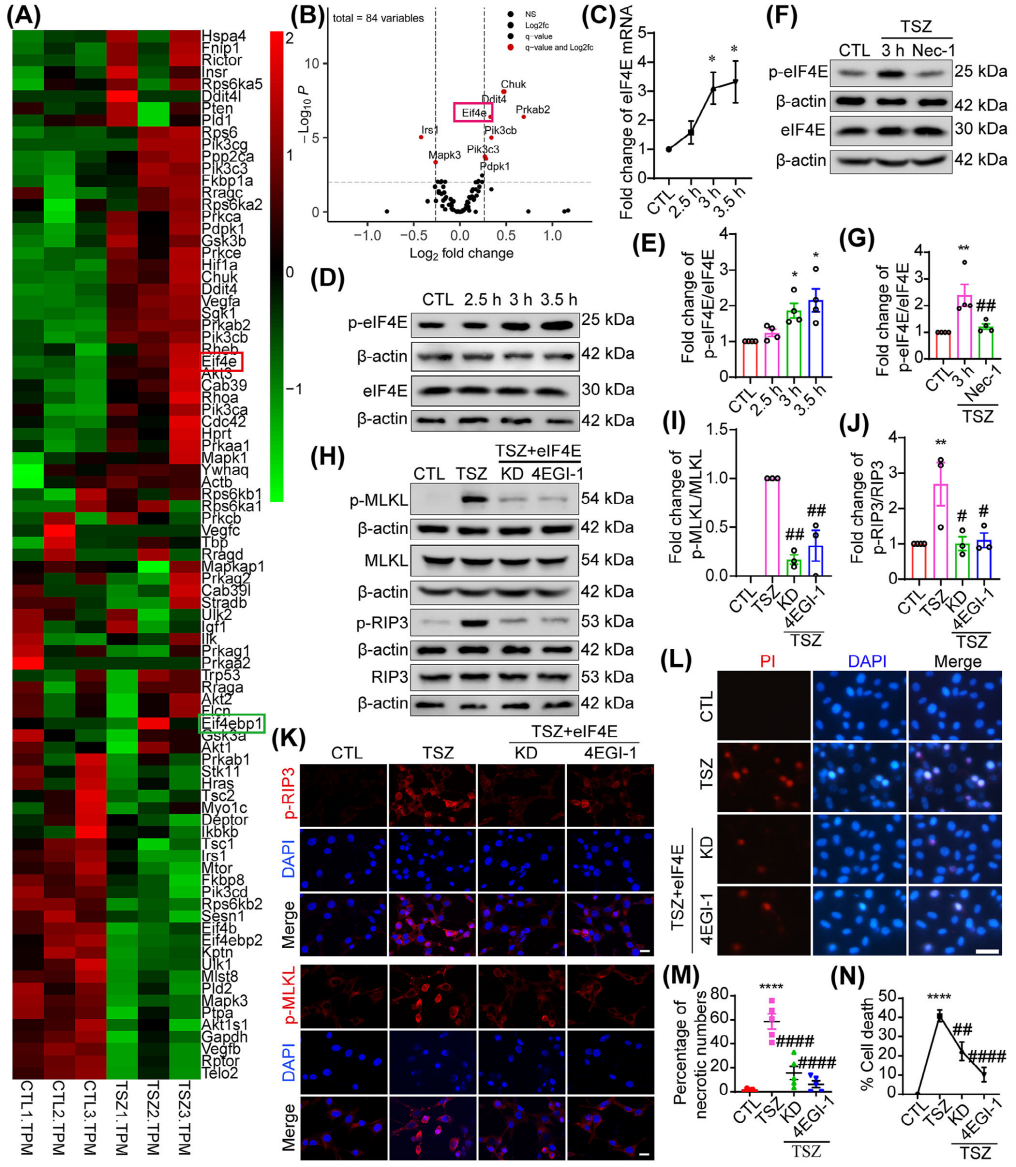
Eukaryotic translation initiation factor 4E (eIF4E) knockdown and inhibition decrease necroptosis. (A) Heat map of RNA sequencing (RNA‐Seq), performed by BGI, of HT22 cells focused on the mammalian target of rapamycin (mTOR) pathway after TNF‐α, Smac mimics, and Z‐VAD‐FMK (TSZ) treatment. (B) Volcano plot, performed by BGI, showing differentially expressed mRNAs in the mTOR pathway. (C) Relative levels of eIF4E mRNA selected from the RNA‐Seq, *n* = 4. (D, F, H) Western blot showing the levels of p‐eIF4E/eIF4E expression in HT22 cells following TSZ treatment or pretreatment with Nec‐1 before TSZ treatment. p‐receptor‐interacting protein‐3 (RIP3)/RIP3 and p‐mixed lineage kinase domain‐like protein (MLKL)/MLKL expression in HT22 cells pretreated with eIF4E siRNA and inhibitor before TSZ treatment. (E, G, I, J) Statistical analysis results of p‐eIF4E/eIF4E, p‐RIP3/RIP3, and p‐MLKL/MLKL expression. (K) Immunofluorescence staining of p‐RIP3 and p‐MLKL in HT22 cells pretreated with eIF4E siRNA and inhibitor before TSZ treatment. Scale bar: 20 µm. (L) eIF4E siRNA‐ and inhibitor‐pretreated HT22 cells were stained with propidium iodide (PI; red) after TSZ treatment. Nuclei were labeled with DAPI (blue). Scale bar: 50 µm. (M) Number of PI‐positive necrotic cells. (N) The percentage of necrotic cells was determined by lactate dehydrogenase (LDH) assay, *n* = 4. *p < 0.05, **p < 0.01, ****p < 0.001 versus control (CTL) group. *##p *< 0.01, *####p *< 0.001 versus TSZ group. Two‐tailed unpaired Student's *t*‐test (G).

To explore the mechanisms underlying necroptosis, RNA sequencing (RNA‐Seq) was performed to determine the transcriptional signatures of necroptotic HT‐22 cells. A total of 15,169 differentially expressed genes (DEGs) were identified between necroptotic HT‐22 cells and normal HT‐22 cells (Figure S2A,B). Considering the important role of the mTOR pathway in necroptosis, the mTOR‐related pathway was evaluated (Figure 2A,B). The top 10 most altered molecules related to the mTOR pathway were chosen, and completed a quantitative polymerase chain reaction (qPCR) assay was performed to verify the RNA‐Seq results (Figures S2C–F and 2C). From this series of formulas, four molecules, eIF4E, VEGFB, MLST8, and SGK1, were identified (Figures S2C–E and 2C), which may participate in necroptosis. As previously mentioned, mTORC1 has three major substrates: 4EBP1/eIF4E, S6K, and ULK1. S6K and RSK1 belong to the AGC kinase superfamily and have similar biological functions. Regarding the participation of ULK1 and RSK1 in necroptosis, based on previous reports, S6K and 4EBP1/eIF4E were hypothesized to play an important role in necroptosis. In this study, the role of 4EBP1/eIF4E, as well as other molecules, in necroptosis was investigated. In addition to the transcriptional level, changes were detected in the eIF4E protein levels. The western blotting results showed that eIF4E expression did not significantly change in necroptotic HT22 cells, whereas the p‐eIF4E levels increased significantly (Figure 2D,E), suggesting that eIF4E activation may be related to the necroptotic process. To determine the role of Nec‐1 in TSZ‐induced eIF4E activation, HT22 cells were pretreated with Nec‐1 before TSZ application. The western blotting results showed that p‐eIF4E expression decreased after Nec‐1 pretreatment (Figure 2F,G), indicating that eIF4E was specifically phosphorylated during necroptosis. In addition, at the end of the observation period, most necroptotic HT22 cells died and were lost. Therefore, a low dose of TSZ was used to injure HT22 cells and eIF4E protein changes were observed over time. The western blotting results showed that eIF4E expression increased at 6 h in necroptotic HT22 cells (Figure S2G,J), which was consistent with the transcriptional results.

To determine the functional role of eIF4E in necroptosis, eIF4E was knocked down using siRNA (Figure S2H,I). In addition, the eIF4E‐specific inhibitor 4EGI‐1 was used to investigate the effects of eIF4E inhibition on the activation of RIP3, MLKL, and necroptosis. As expected, eIF4E knockdown and inhibition significantly decreased RIP3 and MLKL (Figure 2H–J). This result was further confirmed by immunofluorescence (IF), which exhibited an inhibitory effect on p‐RIP3 and p‐MLKL expression after eIF4E knockdown and inhibition compared to the TSZ group (Figure 2K). Furthermore, according to the phase‐contrast images, the PI and LDH results showed that necroptotic HT22 cells were inhibited after eIF4E knockdown and inhibition (Figures 2L–N and S2O). Finally, necroptosis is regarded as an inflammatory reaction, often causing the release of damage‐associated and inflammatory molecular patterns (DAMPs) and proinflammatory cytokines, such as Ccl20, Cxcl8, and Csf1. Furthermore, Ccl20 and Csf1 production was also found to increase after TSZ treatment, which was inhibited by eIF4E knockdown (Figure S2K–N). Thus, eIF4E may be important for the induction of proinflammatory cytokines during necroptosis. These results demonstrate that eIF4E knockdown or inhibition can prevent RIP3 and MLKL activation and effectively inhibit neuronal necroptosis.

### 2.3 4EBP1 overexpression inhibits necroptosis

**FIGURE 3:**
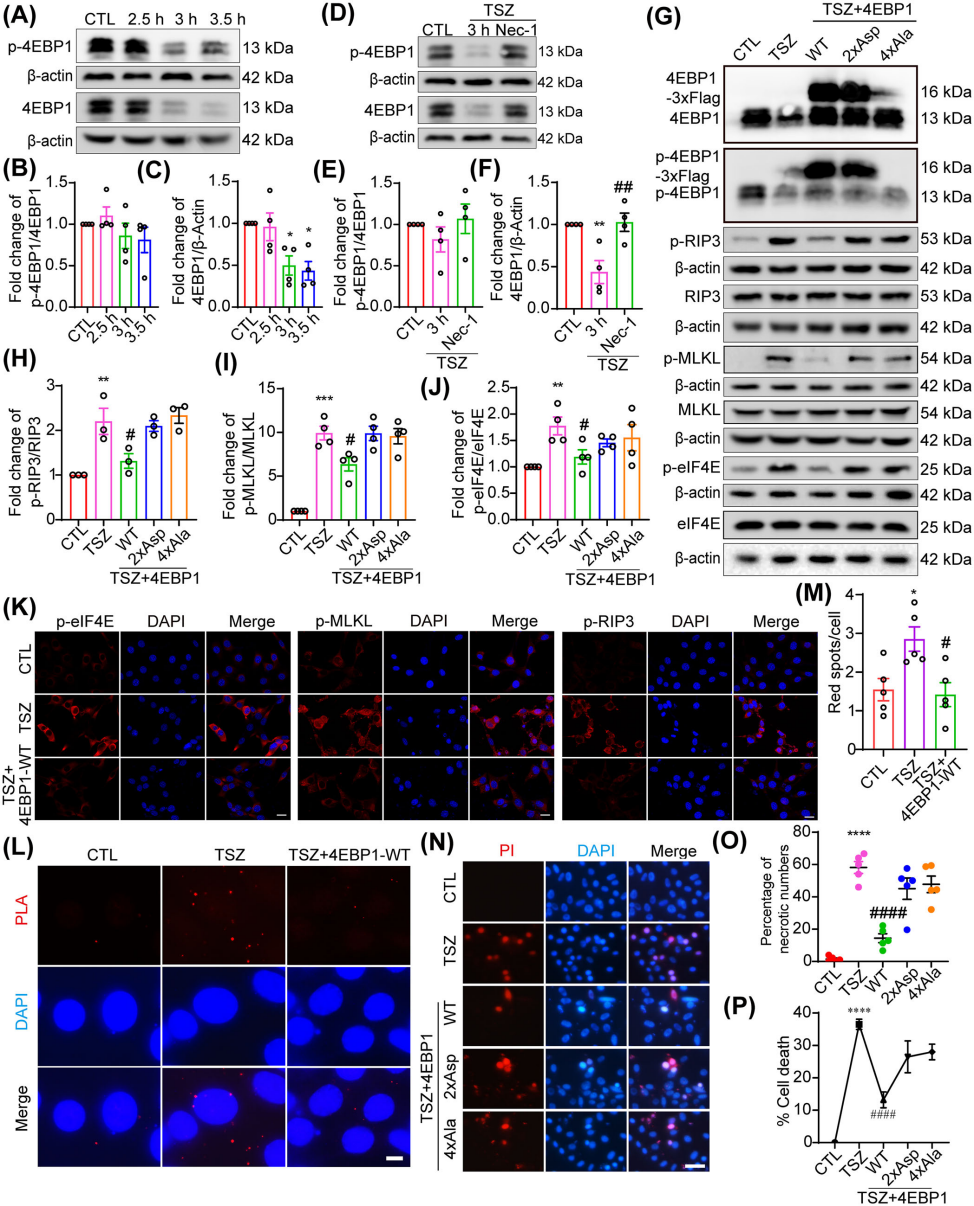
4EBP1 overexpression inhibits necroptosis. (A, D, G) Western blot showing the levels of p‐4EBP1/4EBP1 expression in HT22 cells following TNF‐α, Smac mimics, and Z‐VAD‐FMK (TSZ) treatment or pretreatment with Nec‐1 before TSZ treatment. p‐Eukaryotic translation initiation factor 4E (eIF4E)/eIF4E, p‐receptor‐interacting protein‐3 (RIP3)/RIP3 and p‐mixed lineage kinase domain‐like protein (MLKL)/MLKL expression in HT22 cells pretreated with 4EBP1, 4EBP1‐4xAla and 4EBP1‐2xAsp overexpression before TSZ treatment. (B, C, E, F, H–J) Statistical analysis results of p‐4EBP1/4EBP1, p‐eIF4E/eIF4E, p‐RIP3/RIP3, and p‐MLKL/MLKL expression. (K) Immunofluorescence staining of p‐eIF4E, p‐RIP3, and p‐MLKL in HT22 cells pretreated with 4EBP1 overexpression before TSZ treatment. Scale bar: 20 µm. (L) Positive proximity ligation assay (PLA) dot signals indicate the eIF4G–eIF4E interactions following TSZ treatment or pretreatment with 4EBP1 overexpression before TSZ treatment. Scale bar: 20 µm. (M) Number of PLA dot signals. (N) 4EBP1‐WT, 4EBP1‐4xAla, and 4EBP1‐2xAsp overexpression‐pretreated HT22 cells were stained with propidium iodide (PI; red) after TSZ treatment. Nuclei were labeled with DAPI (blue). Scale bar: 50 µm. (O) Number of PI‐positive necrotic cells. (P) The percentage of necrotic cells was determined by lactate dehydrogenase (LDH) assay, *n* = 4. *p < 0.05, **p < 0.01, ***p < 0.005, ****p < 0.001 versus control (CTL) group. *#p* < 0.05*, ##p* < 0.01 versus TSZ group. Two‐tailed unpaired Student's *t*‐test (E, F, M).

4EBP1 is a translation initiation repressor that binds to and inhibits eIF4E. In this study, changes in and functional roles of 4EBP1 in eIF4E‐mediated necroptosis of HT22 cells were investigated. The western blotting results showed that 4EBP1 and p‐4EBP1 protein expression markedly reduced following TSZ stimulation (Figure 3A,C). In addition, Nec‐1 treatment reversed the TSZ‐induced 4EBP1 and p‐4EBP1 downregulation (Figure 3D,F), indicating that 4EBP1 and p‐4EBP1 downregulation may be specific to the necroptotic process. Phosphorylation of 4EBP1 has an important biological function in 4EBP1 activation and translational repression. The ratio of p‐4EBP1 to 4EBP1 did not change significantly (Figure 3B,E). To eliminate the changes in 4EBP1 activity during apoptosis and necroptosis, HT22 cells were exposed to TS. 4EBP1 phosphorylation was found to increase in apoptotic HT22 cells, which differed from the necroptotic context (Figure S3A–C). Therefore, decreased 4EBP1 protein levels, not the 4EBP1 phosphorylation, were hypothesized to serve as a vital regulator of necroptosis.

To further determine whether 4EBP1 protects against TSZ‐induced necroptosis, the effect of 4EBP1 overexpression on eIF4E, RIP3, and MLKL and changes in necroptosis was examined. The western blotting results showed that p‐eIF4E, p‐RIP3, and p‐MLKL expression was significantly inhibited in the 4EBP1 overexpression (4EBP1‐WT) group, suggesting that 4EBP1 may be related to eIF4E‐mediated necroptosis (Figure 3G–J). This observation was further confirmed by IF, which exhibited an inhibitory effect on p‐eIF4E, p‐RIP3, and p‐MLKL expression after 4EBP1 overexpression compared to the TSZ group (Figure 3K). Hypophosphorylated 4EBP1 (4EBP1‐4xAla) or hyperphosphorylated 4EBP1 (4EBP1‐2xAsp) plays an important role in many biological processes; therefore, 4EBP1‐4xAla and 4EBP1‐2xAsp were overexpressed in HT22 cells. In line with our observation that the ratio of p‐4EBP1 to 4EBP1 did not change during the necroptotic process, both 4EBP1‐4xAla and 4EBP1‐2xAsp overexpression had no significant effect on the changes in p‐eIF4E, p‐RIP3, and p‐MLKL expression (Figure 3G–J). These results indicate that the phosphorylation status of 4EBP1 may not modulate eIF4E, RIP3, or MLKL activation in a necroptotic context. 4EBP1 competitively binds eIF4E and inhibits its interaction with eIF4G. To investigate the effect of 4EBP1 on the eIF4G–eIF4E interaction in the necroptotic process, a proximity ligation assay (PLA) was used in situ to detect the eIF4G–eIF4E interaction. The results showed that the positive PLA dot signals increased in necroptotic HT22 cells (Figure 3L,M), indicating that the eIF4G–eIF4E interaction and its mediated translation of necroptotic factors may be enhanced during necroptosis. Furthermore, PLA signals were inhibited after 4EBP1 overexpression, indicating disruption of the eIF4G–eIF4E interaction and inhibition of necroptotic factor translation (Figure 3L,M). Finally, PI staining and LDH assay showed that necroptotic HT22 cells were inhibited after 4EBP1 overexpression compared to the TSZ group (Figures 3M–O and S3O). While both 4EBP1‐4xAla and 4EBP1‐2xAsp overexpression have no significant effect on necroptotic HT22 cells (Figure 3N–P), Ccl20 and Csf1 expression induced by TSZ was also inhibited by 4EBP1 overexpression but not by 4EBP1‐4xAla and 4EBP1‐2xAsp overexpression (Figure S3D,E). These results indicate that 4EBP1 overexpression prevents the activation of eIF4E, RIP3, MLKL, and neuronal necroptosis.

In some physiological or pathological states, eIF4E regulates 4EBP1 protein levels. Therefore, whether eIF4E modulates 4EBP1 levels in a necroptotic context was investigated. The western blotting results showed that 4EBP1 and p‐4EBP1 were unchanged after eIF4E knockdown and inhibition, suggesting that 4EBP1 is not a downstream molecule of eIF4E in the necroptotic process (Figure S3F,G). In addition, as previously mentioned, this suggests that S6K may also be a regulator of necroptosis and verified this in another study (unpublished data). Based on reports that 4EBP1 may regulate S6K activity, whether 4EBP1 regulates necroptosis via S6K and RSK was investigated. The western blotting results showed that S6K and RSK were activated in necroptotic HT22 cells (Figure S3H–J and unpublished data). The overexpression of 4EBP1, 4EBP1‐4xAla, and 4EBP1‐2xAsp had no significant effect on S6K and RSK activities compared to the TSZ group (Figure S3K–M and unpublished data), indicating that 4EBP1 participates in the regulation of necroptosis without S6K or RSK.

### 2.4 4EBP1 is degraded through ubiquitination in necroptosis

**FIGURE 4:**
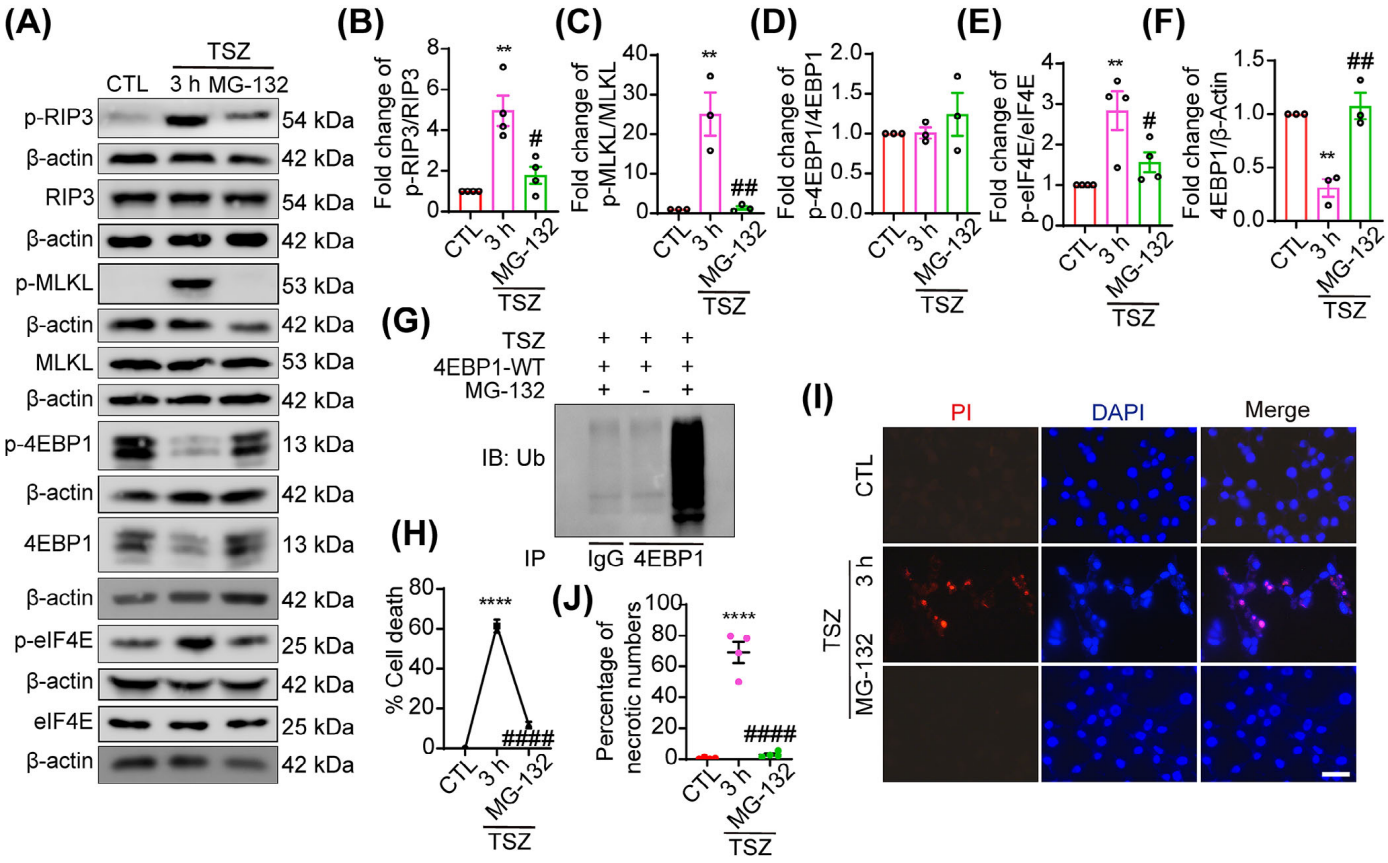
4EBP1 is degraded through ubiquitination in necroptosis. (A) Western blot showing the levels of p‐4EBP1/4EBP1, p‐eukaryotic translation initiation factor 4E (eIF4E)/eIF4E, p‐receptor‐interacting protein‐3 (RIP3)/RIP3, and p‐mixed lineage kinase domain‐like protein (MLKL)/MLKL expression in HT22 cells following TNF‐α, Smac mimics, and Z‐VAD‐FMK (TSZ) treatment or pretreatment with MG‐132 before TSZ treatment. (B–F) Statistical analysis results of p‐4EBP1/4EBP1, p‐eIF4E/eIF4E, p‐RIP3/RIP3, and p‐MLKL/MLKL expression. (G) Co‐immunoprecipitation assay showing the binding of 4EBP1 to ubiquitin. (H) The percentage of necrotic cells was determined using the lactate dehydrogenase (LDH) assay, *n* = 4. (I) MG‐132 pretreated HT22 cells were stained with propidium iodide (PI; red) after TSZ treatment. The nuclei were labeled with DAPI (blue). Scale bar: 50 µm. (J) Number of PI‐positive necrotic cells. **p < 0.01, ****p < 0.001 versus control (CTL) group*. #p *< 0.05, *##p *< 0.01, *####p *< 0.001 versus TSZ 3 h group. Two‐tailed unpaired Student's *t*‐test (B–F, H, J).

The western blotting results showed that the 4EBP1 protein levels were markedly reduced at 3 h following TSZ treatment (Figure 3A,C). Based on these findings, the mechanism underlying the decrease in 4EBP1 expression following TSZ injury was evaluated. qPCR showed that the mRNA level of 4EBP1 was unaffected in TSZ‐treated HT22 cells (Figure S3N), strongly suggesting that post‐transcriptional mechanisms primarily regulate 4EBP1 levels. Furthermore, MG132, a proteasome inhibitor, restored the 4EBP1 protein levels, suggesting that 4EBP1 is degraded through the ubiquitination pathway (Figure 4A,F). To directly determine whether TSZ‐induced 4EBP1 ubiquitination occurred during necroptosis, co‐immunoprecipitation (Co‐IP) assay was used to examine the binding of 4EBP1 with ubiquitin. Ubiquitination analysis revealed that MG132 treatment increased the binding of 4EBP1 to ubiquitin, indicating that 4EBP1 ubiquitination is involved in TSZ‐induced necroptosis (Figure 4G). These data suggest that decreased 4EBP1 protein levels after TSZ injury are likely due to activation of the ubiquitin pathway. To further explore the possibility that changes in necroptotic HT22 cells are related to the ubiquitination pathway, HT22 cells were treated with a proteasome inhibitor. The western blotting results showed that eIF4E, RIP3, and MLKL activation was inhibited after MG132 treatment (Figure 4A–E). In addition, the PI and LDH results indicated that MG132 treatment almost completely protected HT22 cells from death (Figure 4H–J). These results suggest that 4EBP1 may be degraded in necroptotic HT22 cells via the ubiquitin‐proteasome pathway.

### 2.5 RIP3 and MLKL regulate necroptosis through the 4EBP1–eIF4E pathway

**FIGURE 5:**
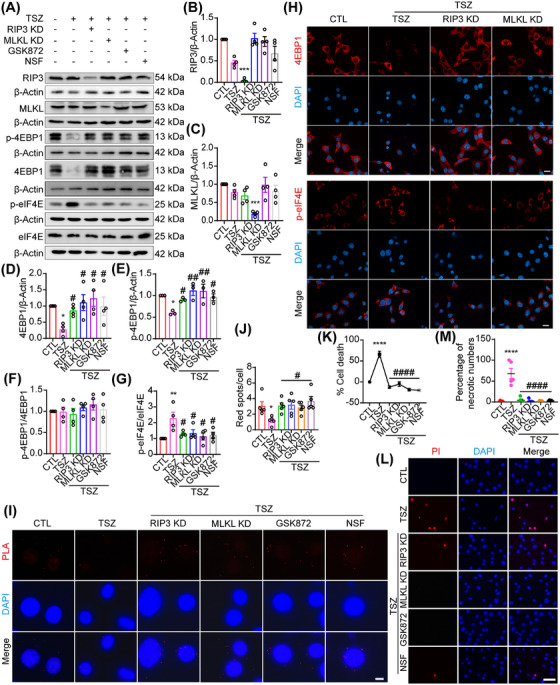
Receptor‐interacting protein‐3 (RIP3) and mixed lineage kinase domain‐like protein (MLKL) regulate necroptosis through the 4EBP1–eukaryotic translation initiation factor 4E (eIF4E) pathway. (A) Western blot showing the levels of p‐4EBP1/4EBP1, p‐eIF4E/eIF4E, RIP3, and MLKL expression in HT22 cells pretreated with RIP3/MLKL siRNAs and inhibitors before TNF‐α, Smac mimics, and Z‐VAD‐FMK (TSZ) treatment. (B–G) Statistical analysis of p‐4EBP1/4EBP1, p‐eIF4E/eIF4E, RIP3, and MLKL expression. (H) Immunofluorescence staining of 4EBP1 and p‐eIF4E in HT22 cells pretreated with RIP3 and MLKL siRNAs before TSZ treatment. Scale bar: 20 µm. (I) Positive proximity ligation assay (PLA) dot signals indicate the 4EBP1–eIF4E interactions following TSZ treatment or pretreatment with RIP3/MLKL siRNAs and inhibitors before TSZ treatment. Scale bar: 20 µm. (J) Number of PLA dot signals. (K) The percentage of necrotic cells was determined by lactate dehydrogenase (LDH) assay, *n* = 4. (L) RIP3/MLKL knockdown and inhibition pretreated HT22 cells were stained with propidium iodide (PI; red) after TSZ treatment. Nuclei were labeled with DAPI (blue). Scale bar: 50 µm. (M) Number of PI‐positive necrotic cells. *p < 0.05, **p < 0.01, ***p < 0.005, ****p < 0.001 versus control (CTL) group. *#p *< 0.05, *##p *< 0.01, *####p *< 0.001 versus TSZ group.

Intriguingly, the western blotting results (Figures 1, 2, and 3A,C) showed changes in RIP3 and MLKL activation before changes in 4EBP1 degradation and eIF4E activation. To further explore the regulatory relationship between the RIP3–MLKL and 4EBP1–eIF4E pathways, HT22 cells were pretreated with RIP3 and MLKL siRNAs and inhibitors, which increased the levels of 4EBP1 and p‐4EBP1 and attenuated the expression of p‐eIF4E compared with the TSZ groups (Figure 5A–G). Consistent with the western blotting results, the IF assay showed an enhanced effect on 4EBP1 protein and an inhibitory effect on p‐eIF4E expression after RIP3 and MLKL knockdown, compared to TSZ treatment (Figure 5H). These results demonstrated that RIP3 and MLKL knockdown or inhibition can regulate changes in the 4EBP1–eIF4E pathway. Since 4EBP1 binds to and inhibits eIF4E to suppress translation, a PLA assay was used to detect the 4EBP1–eIF4E interaction. The results showed that the positive PLA signals decreased in necroptotic HT22 cells, indicating that the 4EBP1–eIF4E interaction was inhibited, and necroptotic factor translation was enhanced during necroptosis (Figure 5I,J). Furthermore, PLA signals increased after RIP3 and MLKL knockdown and inhibition, indicating the enhancement of the 4EBP1–eIF4E interaction and the inhibition of necroptotic factor translation (Figure 5I,J). Finally, the PI staining and LDH assay results indicated that necroptotic HT22 cells were inhibited after RIP3 and MLKL knockdown and inhibition compared with the TSZ group (Figure 5K–M). In addition, TSZ‐induced Ccl20 and Csf1 expression was inhibited by RIP3 and MLKL knockdown and inhibition (Figure S4A,B). Taken together, these results indicate that the RIP3–MLKL pathway contributes to changes in the 4EBP1–eIF4E pathway during necroptosis, suggesting a positive feedback loop between the RIP3–MLKL and 4EBP1–eIF4E pathways.

**FIGURE 6:**
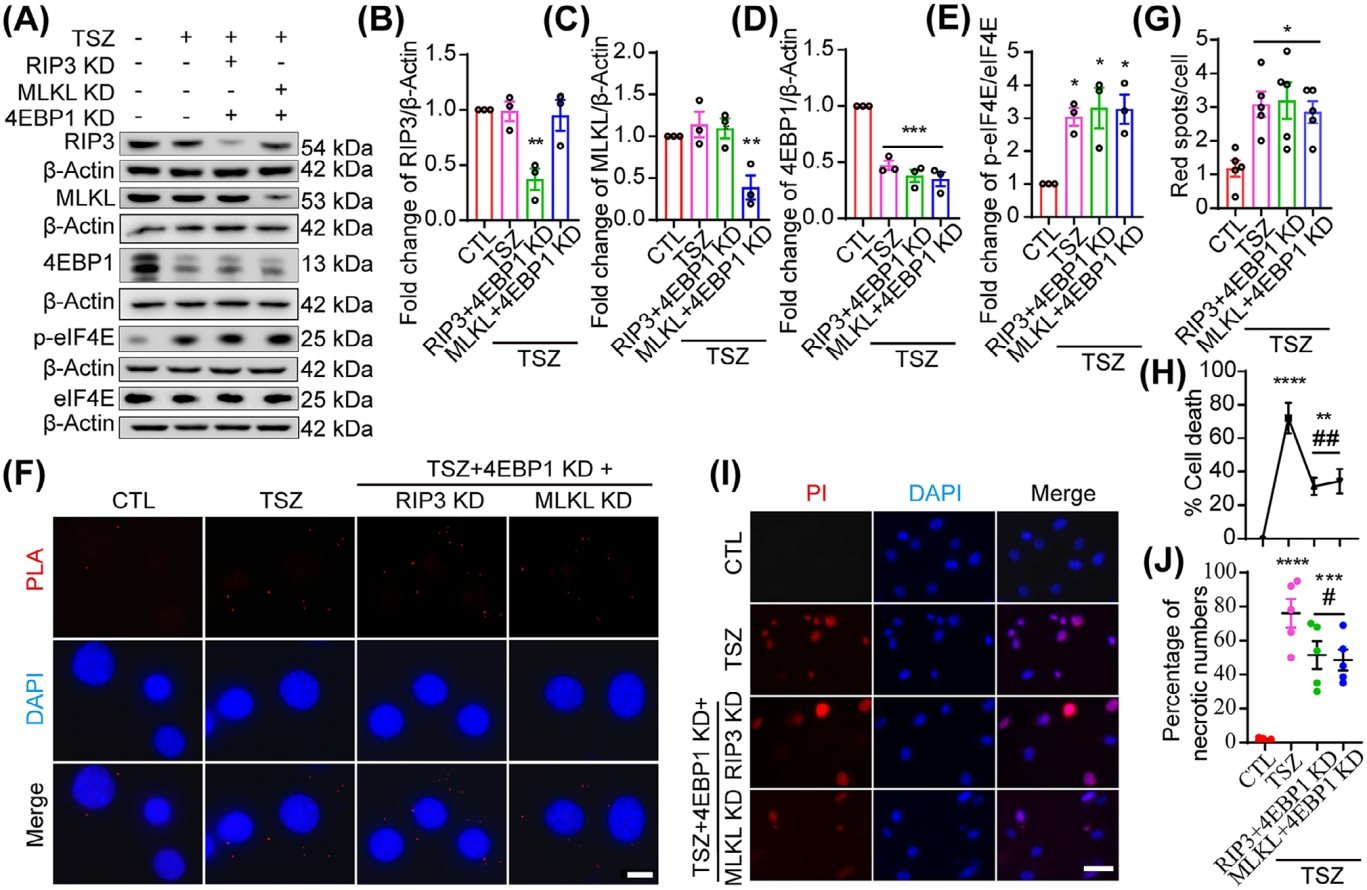
Receptor‐interacting protein‐3 (RIP3) and mixed lineage kinase domain‐like protein (MLKL) regulate necroptosis through the 4EBP1–eukaryotic translation initiation factor 4E (eIF4E) pathway. (A) Western blot showing the levels of 4EBP1, RIP3, MLKL, and p‐eIF4E/eIF4E expression in HT22 cells pretreated with 4EBP1 and RIP3/MLKL siRNAs before TNF‐α, Smac mimics, and Z‐VAD‐FMK (TSZ) treatment. (B–E) Statistical analysis of 4EBP1, RIP3, MLKL, and p‐eIF4E/eIF4E expression. (F) Positive proximity ligation assay (PLA) dot signals indicate the eIF4E–eIF4G interactions following TSZ treatment or pretreatment with 4EBP1 and RIP3/MLKL siRNAs before TSZ treatment. Scale bar: 20 µm. (G) Number of PLA dot signals. (H) The percentage of necrotic cells was determined by lactate dehydrogenase (LDH) assay, *n* = 4. (I) 4EBP1 and RIP3/MLKL double knockdown pretreated HT22 cells were stained with propidium iodide (PI; red) after TSZ treatment. Nuclei were labeled with DAPI (blue). Scale bar: 50 µm. (J) Number of PI‐positive necrotic cells. *p < 0.05, **p < 0.01, ***p < 0.005, ****p < 0.001 versus control (CTL)Homologous to the C‐terminus of E6AP (HECT) and RCC1‐like domain (RLD) group. *#p *< 0.05, ##*p *< 0.01 versus TSZ group.

To further investigate the roles of RIP3 and MLKL in the regulation of necroptosis via the 4EBP1–eIF4E pathway, a rescue assay was performed. HT22 cells were first transduced with RIP3 or MLKL siRNA and then co‐transduced with 4EBP1 siRNA (Figure 6A–E). Finally, RIP3 and 4EBP1 double‐KD cells, or MLKL and 4EBP1 double‐KD HT22 cells, were exposed to TSZ (Figure 6A–E). TSZ was found to induce necroptosis in RIP3 and 4EBP1 double KD cells or MLKL and 4EBP1 double KD HT22 cells (Figure 6I,J), which was almost absent in RIP3 and MLKL KD cells (Figure 5K–M), indicating that 4EBP1 may be a downstream molecule of the RIP3–MLKL pathway. Specifically, eIF4E activation and eIF4E–eIF4G interaction in TSZ‐exposed RIP3 and 4EBP1 double‐KD cells or TSZ‐exposed MLKL and 4EBP1 double‐KD HT22 cells was examined. As a result, TSZ‐exposed cells, and TSZ‐exposed RIP3 and 4EBP1 double KD cells, and TSZ‐exposed MLKL and 4EBP1 double KD were found to induce significantly higher p‐eIF4E levels and eIF4E–eIF4G interactions compared to the control group (Figures 5I,J and 6F,G), demonstrating that TSZ stimulation and 4EBP1 silencing may be have similar effects on the eIF4E‐mediated necroptotic process. Notably, TSZ only induced partial RIP3 and 4EBP1 double KD cells, or partial TSZ‐exposed MLKL and 4EBP1 double KD cells underwent necroptosis compared with TSZ, TSZ‐exposed RIP3, or MLKL KD cell groups (Figures 5K–M and 6I,J), indicating that, except for the 4EBP1–eIF4E pathway, other key molecules participate in regulating necroptosis downstream of the RIP3–MLKL pathway.

### 2.6 Pharmaceutical inhibition of eIF4E, RIP3, and MLKL indicates a feedback loop between eIF4E and RIP3–MLKL pathways in mice MCAO model

**FIGURE 7:**
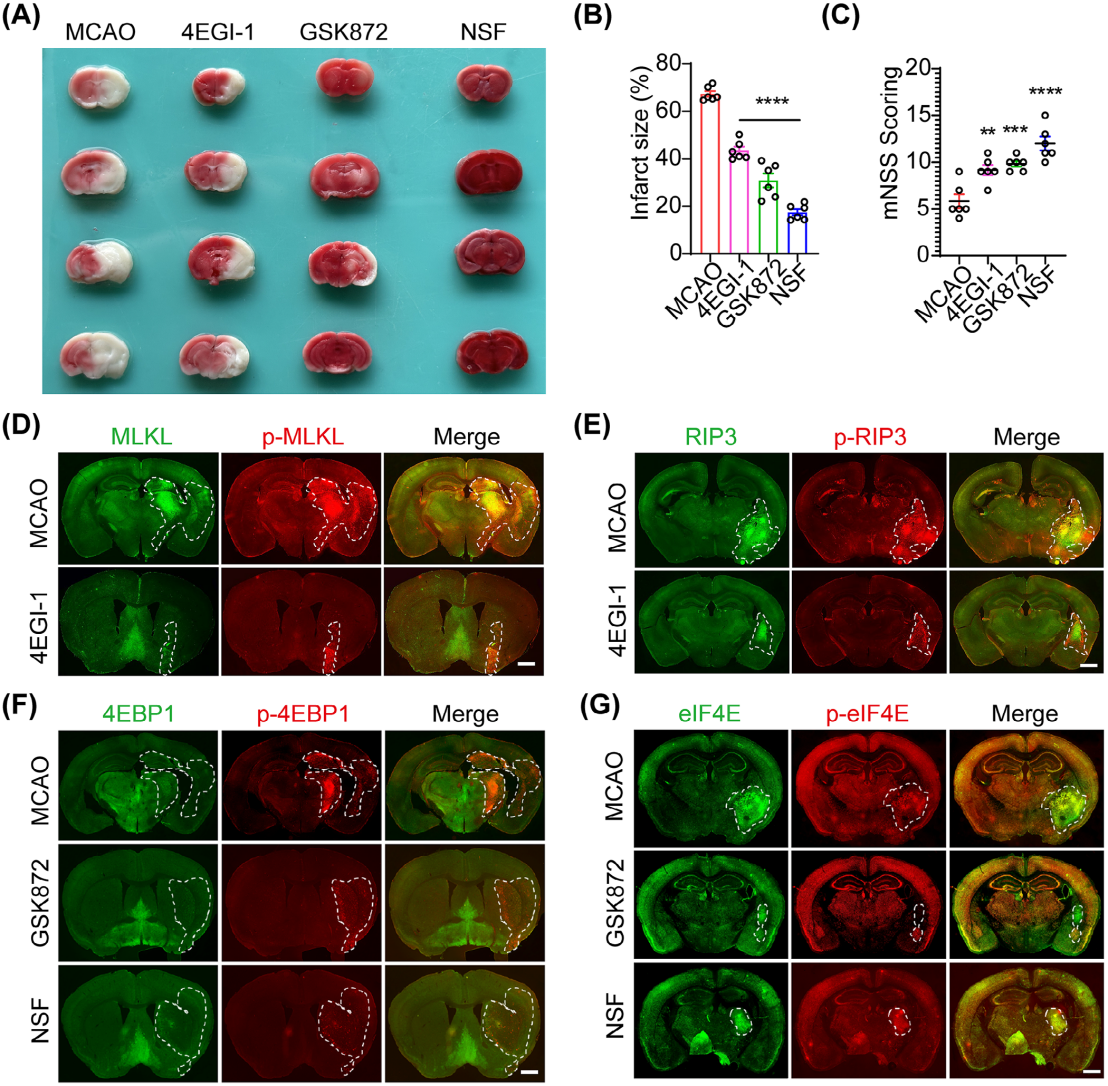
Pharmaceutical inhibition of eukaryotic translation initiation factor 4E (eIF4E), receptor‐interacting protein‐3 (RIP3), and mixed lineage kinase domain‐like protein (MLKL) indicates a feedback loop between the eIF4E and RIP3–MLKL pathways in mice middle cerebral artery occlusion (MCAO) model. (A) 2,3,5‐Triphenyltetrazolium chloride (TTC) staining showing the ischemic area in mice brains pretreated with eIF4E, RIP3, and MLKL inhibitors before MCAO injury. (B) Statistical analysis of TTC staining. (C) Modified neurological severity scores (mNSS) scoring showing neurological function in mice brains pretreated with eIF4E, RIP3, and MLKL inhibitors before MCAO injury. (D, E) Immunofluorescence staining of p‐RIP3/RIP3 and p‐MLKL/MLKL in mice brain pretreated with eIF4E inhibitor before MCAO injury. (F, G) Immunofluorescence staining of p‐4EBP1/4EBP1 and p‐eIF4E/eIF4E in mice brain pretreated with RIP3 and MLKL inhibitor before MCAO injury. ***p < 0.01, **p < 0.005, ****p < 0.001 versus MCAO group. Scale bar: 1 mm.

Given reports that the inhibition of RIP3 and MLKL attenuate necroptosis in the MCAO model and the in vitro results of this study showing a positive feedback loop between RIP3–MLKL and 4EBP1–eIF4E pathways in the necroptotic process, the in vitro results were further verified using a MCAO model. After unilateral intracerebroventricular injection of eIF4E, RIP3, and MLKL inhibitors, ischemic infarct size was quantified by 2,3,5‐triphenyltetrazolium chloride (TTC) staining and neurological function was evaluated by modified neurological severity scores (mNSS) scoring (Figure 7A–C). The eIF4E, RIP3, and MLKL inhibitor groups showed significantly reduced ischemic areas and neurological deficit scores compared to the MCAO group (Figure 7A–C). Immunofluorescence staining showed that the volume and intensity of p‐4EBP1, p‐eIF4E/eIF4E, p‐RIP3/RIP3, and p‐MLKL/MLKL were increased in the damaged area compared to the contralateral side (Figure 7D–G). Mice were then intracerebroventricularly injected with eIF4E, RIP3, or MLKL inhibitors before MCAO injury. The volume and intensity of both p‐MLKL/MLKL (Figure 7D) and p‐RIP3/RIP3 (Figure 7E) staining decreased after eIF4E inhibition by 4EGI‐1, indicating that eIF4E regulates the RIP3–MLKL pathway in the MCAO model. Furthermore, immunofluorescence staining showed that the volume and intensity of both p‐eIF4E/eIF4E (Figure 7G) and p‐4EBP1 (Figure 7F) decreased after RIP3 and MLKL inhibition using GSK872 and Necrosulfonamide (NSF), indicating that the RIP3–MLKL pathway regulates the 4EBP1–eIF4E pathway in the MCAO model. It should be noted that the results of the in vivo studies indicated that the expression of 4EBP1 did not change after MCAO injury (Figure 7F), whereas the p‐4EBP1 levels were increased (Figure 7F), which differed from the in vitro results. However, the ratio of p‐4EBP1 to 4EBP1 increased in vivo, indicating an attenuated inhibitory effect on eIF4E, which was consistent with the in vitro findings. These in vivo results indicate that inhibition of eIF4E, RIP3, and MLKL provides powerful neuroprotective effects against MCAO injury by modulating the positive feedback loop between the RIP3–MLKL and 4EBP1–eIF4E pathways in the MCAO model.

## 3 DISCUSSION

The findings revealed a noncanonical and positive amplification loop between the RIP3–MLKL and 4EBP1–eIF4E pathways in neuronal necroptosis, which differs from the traditional translation function of the 4EBP1–eIF4E pathway. The 4EBP1–eIF4E pathway is activated during neuronal necroptosis. 4EBP1 overexpression and eIF4E inhibition blocked necroptosis by regulating the RIP3–MLKL pathway. In addition, the 4EBP1–eIF4E pathway regulates the RIPK3‐MLKL pathway via a positive feedback circuit in which the RIPK3‐MLKL pathway stimulates the 4EBP1–eIF4E pathway by degrading 4EBP1 via the ubiquitin system, which in turn increases the activity of RIP3 and MLKL. Finally, excessive eIF4E activation, possibly derived from this circuit, induces chemokines and cytokines that may contribute to necroptotic inflammation.

In a previous observational study, we found that eIF4E increased and 4EBP1 decreased, accompanied by necroptosis in L929 cells.^28^ However, the specific regulatory mechanism between RIP3–MLKL and 4EBP1–eIF4E during necroptosis remained unknown. In the present study, a positive regulatory mechanism was revealed between the 4EBP1–eIF4E pathway and neuronal necroptosis, wherein both 4EBP1 overexpression and eIF4E inhibition blocked the development of necroptosis. The traditional view is that the initiation of protein synthesis is controlled by the eIF4F complex, which is composed of the cap‐binding protein eIF4E, the RNA helicase eIF4A, and the scaffolding protein eIF4G, which recruits mRNA to the ribosome.^22^ The hyperphosphorylation of 4EBP1 disrupts its interaction with eIF4E, which inhibits cap‐dependent translation.^29^ As necroptosis is an inflammatory process that often causes the release of proinflammatory cytokines, the production of Ccl20 and Csf1, which are two common indices in necroptotic research, was measured.^30^, ^31^, ^32^ The 4EBP1–eIF4E pathway was found to modulate the production of Ccl20 and Csf1 during neuronal necroptosis. Increased protein synthesis is associated with neuronal toxicity or dysfunction in some neurodegenerative disorders, such as stroke, Huntington's disease (HD), and Alzheimer's disease (AD).^33^, ^34^, ^35^ 4EBP1 phosphorylation is altered in the striatum and increases the formation of the eIF4F complex, consequently leading to exaggerated translation in Parkinson's disease (PD).^36^, ^37^ In AD mouse brains, increased p‐4EBP and decreased total 4EBP1 levels correlated with tau pathology, which is similar to our results.^38^ The pharmacological normalization of protein synthesis in AD mice ameliorates motor disturbances.^39^, ^40^ Thus, the results of the present study support the view that altered 4EBP1–eIF4E function could be a common mechanism for dysregulated protein synthesis in many neurodegenerative disorders. These findings have important implications for translational control, as 4EBP1 downregulation and eIF4E activation under detrimental conditions promote the production of necroptotic factors. Consistent with the role of the 4EBP1–eIF4E pathway in necroptosis, the activated 4EBP1–eIF4E pathway can also induce apoptosis owing to its high and constant metabolic rate and energy consumption.^33^, ^41^ Other studies have indicated that when encountering stress signals or cancers, elevated 4EBP1–eIF4E activation may inhibit apoptosis.^42^, ^43^ These differences may be related to different microenvironments and diseases, suggesting selective translation of key proteins and perversion of cellular energy utilization during specific pathophysiological processes.

This study found that the 4EBP1 protein levels, rather than mRNA levels, decreased during TSZ‐induced necroptosis, suggesting that 4EBP1 may change at the post‐transcriptional level during this process. These findings indicated that 4EBP1‐binding ubiquitin significantly increased during necroptosis, indicating a possible role of 4EBP1 ubiquitination in the pathogenesis of necroptosis. However, the degradation pathway responsible for 4EBP1 remained unclear. Previous studies have reported a series of findings concerning the degradation of 4EBP1, including degradation by the proteasome in herpes simplex virus‐1 (HSV‐1)‐infected cells.^44^ Impairment of 4EBP1 stability promotes replication and latent infections of HSV‐1.^44^ Furthermore, their data suggested that the phosphorylation of 4EBP1 was more sensitive to proteasomal degradation.^44^ However, other studies have demonstrated that 4EBP1 ubiquitination serves as a homeostatic mechanism to maintain translation at normal levels commensurate with cell survive.^45^, ^46^ For example, under physiological stresses such as amino acid starvation, DNA damage, or the activation of p53, the 4EBP1 protein might be more stable.^46^, ^47^ 4EBP1 triggers apoptosis in response to glucose deprivation in the central region of non–small‐cell lung cancer (NSCLC).^47^ In NSCLC tissues, glucose starvation (GS) decreased the ubiquitination of 4EBP1, leading to high and stable 4EBP1 levels.^47^ This study also identified Homologous to the C‐terminus of E6AP and RCC1‐like domain‐containing E3 ubiquitin protein ligase 5 (HERC5) as an E3 ligase that targets 4EBP1 for degradation, which could be prevented by GS in NSCLC.^47^ Moreover, 4EBP1 knockdown increased tumor volume and weight in xenograft models by inhibiting apoptosis in the central region of tumor,^47^ indicating the different roles of 4EBP1 in cell survival. Although the present study indicates that 4EBP1 ubiquitination may play an important role in necroptosis, the ubiquitin ligase in 4EBP1 ubiquitination has yet to be determined. This will be the topic of future research.

In the present study, changes were observed in the RIP3–MLKL pathway prior to changes in the 4EBP1–eIF4E pathway. These findings suggest that the RIP3–MLKL pathway is found upstream of the 4EBP1–eIF4E pathway. Specifically, deficiency or inhibition of RIP3 and MLKL was found to recover the 4EBP1 protein levels, which may be degraded by ubiquitin. Furthermore, a rescue assay was performed, with the results indicating that TSZ could induce necroptosis in RIP3/MLKL and 4EBP1 double‐KD cells, which almost disappeared in RIP3/MLKL KD cells, indicating that 4EBP1 is a downstream molecule of the RIP3–MLKL pathway. During our study, Li et al. independently reported that in triple‐negative breast cancer (TNBC), RIP1‐dependent necroptosis promoted vasculogenic mimicry formation by increasing the expression of eIF4E, which is similar to our results.^48^ However, the role of phosphorylated eIF4E and its endogenous inhibitor, 4EBP1, in RIP3–MLKL pathway‐mediated necroptosis has not yet been elucidated. Our study demonstrated that activation of the RIP3–MLKL pathway could exacerbate and sustain necroptotic inflammation through the 4EBP1–eIF4E pathway. Other studies have indicated that the formation of a positive feedback circuit that enhances and sustains a specific response is a widely involved inflammatory process.^49^ Notably, TSZ only induces partial RIP3/MLKL and 4EBP1 double KD cells that undergo necroptosis compared to the TSZ, TSZ‐exposed RIP3, and MLKL KD cell groups, indicating that, except for the 4EBP1–eIF4E pathway, other key molecules participate in regulating necroptosis downstream of the RIP3–MLKL pathway. Finally, in vivo studies showed significantly reduced ischemic areas and neurological deficit scores in the eIF4E, RIP3, and MLKL inhibitor groups compared to those in the MCAO group, indicating that inhibition of eIF4E, RIP3, and MLKL provides powerful neuroprotective effects against MCAO injury. These findings suggest that the positive feedback loop between the 4EBP1–eIF4E and RIP3–MLKL pathways might serve as an important regulator of inflammatory cascades and explain necroptosis.

Stroke is a common nervous system disorder with a relatively high prevalence, and the current pathophysiological mechanism remains poorly defined, leading to the fact that current therapies for stroke focus on controlling signs and symptoms. In the present study, the essential role of the 4EBP1–eIF4E and RIP3–MLKL pathways in the pathogenesis of necroptosis was revealed by employing both gene overexpression/knockdown and pharmacological inhibition methods, strongly indicating that targeting 4EBP1–eIF4E may be a promising option for the treatment of necroptosis‐mediated diseases. However, the results of this study are primarily from TSZ injured cell model, which have not been verified in ischemic cell models and clinical samples related to stroke disease. So the clinical significance of this research for stroke therapy remains unclear, which should be further clarified.

## 4 CONCLUSION

Our findings revealed a noncanonical and positive amplification loop between “RIP3–MLKL” and “4EBP1–eIF4E” pathways in neuronal necroptosis. It is indicated that 4EBP1–eIF4E pathway regulates the RIPK3‐MLKL pathway via a positive feedback circuit in which the RIPK3‐MLKL pathway stimulates the 4EBP1–eIF4E pathway by degrading 4EBP1 via the ubiquitin system, which in turn increases the activity of RIP3 and MLKL.

## 5 METHODS

### 5.1 HT22 cell line cultures, in vitro necroptotic/apoptotic models, and drugs application

HT22 neuronal cell line was from American Type Culture Collection (ATCC) and cultured in Dulbecco's modified Eagles's medium (DMEM) with 10% fetal bovine serum (FBS; Gibco). Cells were cultured under 37°C and 5% CO_2_. Commercial TSZ or TS kits (Beyotime) was applied and maintained for different observed hours to induce the necroptotic or apoptotic models. For inhibitors application, Nec‐1 (0.49 µM), 4EGI‐1 (25 µM), MG‐132 (1.2 µM), GSK872 (1 µM), and NSF (5 µM; MedChemExpress) were added 30 min before TSZ application.

### 5.2 Mice MCAO model, drugs application, and tissue preparation

All animal experiments were approved by the animal research committee of The Second Xiangya Hospital of Central South University. APPROVAL NUMBER: 2021136. Specifically, Male C57/BL6 mice (8 weeks, 30 g) were randomly assigned to MCAO group or inhibitor treated groups. Transient MCAO model was performed as previously described. Briefly, mice were anesthetized by isoflurane. Then, a silicon suture (Doccol) was placed into the middle cerebral artery to make blood occlusion for 60 min. During MCAO injury, the regional cerebral blood flow was measured. The mice with blood flow that reduced more than 50% flow were included for the next experiments. For inhibitor treated groups, a volume of 1.5 µL (20 mM) 4EGI‐1, GSK872 or NSF was stereotaxically injected into the ventricle (anterior–posterior, 0.8 mm; medio‐lateral, 1.4 mm; dorso‐ventral, 3.6 mm) 30 min before MCAO. After 3 days reperfusion, mice were perfused with paraformaldehyde (PF). The brains were removed, postfixed in PF, equilibrated in sucrose solutions, embedded by OCT Tissue‐Tek (Sakura), performed frozen sections (30 µm) and stored at −20°C.

### 5.3 TTC staining

TTC staining was used to evaluate the infarct volume after MCAO. Briefly, the mice brains were removed and cut into 2 mm sections. Then, the sections were stained with TTC dye for 15 min (Beyotime) and fixed in PF. Last, the sections were captured and the infarct volume was measured by using Image J (NIH). The percentage of infarct volume were determined by infarct area/sections area.

### 5.4 mNSS scoring

mNSS scoring was performed by blinded independent persons to evaluate the neurological functions after MCAO. The score was divided into 0–18 and included sensory, motor, reflex, and balance tests, with normal mice scored as 18 and maximal neurological deficit scored as 0. Cumulative scores of 1–6, 6–12, and 13–18 indicate severe, moderate, and mild deficit, respectively.

### 5.5 PI staining

At the observed time points, HT22 cells were stained with PI and then washed with phosphate‐buffered saline (PBS). Next, cell cultures were covered by mounting medium contained with 4',6‐diamidino‐2‐phenylindole (DAPI) (Beyotime) and imaged by fluorescence microscope (Zeiss). The percentage of PI‐positive necrotic cells were counted by Image J (NIH).

### 5.6 LDH release assay

The LDH assay was performed as the instructions (Beyotime). Briefly, the cell supernatants were collected by centrifugation and added to the pretreated reagent mixture. After incubated together, the absorbance was detected at 490 nm wavelength. The percentage of necrosis were determined by (the absorbance of treated cells minus control cells)/(LDH releasing reagent treated cells minus control cells).

### 5.7 Western blotting

HT22 cells were lysed with radio immunoprecipitation assay (RIPA) lysis containing protease and phosphatase inhibitors (Beyotime). A total of 10 µg total proteins were loaded and separated by sodium dodecyl‐sulfate polyacrylamide gel electrophoresis (SDS‐PAGE) gel, and transferred to polyvinylidene difluoride (PVDF) membrane (Merk Millipore). The membrane was blocked with nonfat milk, incubated with primary antibodies: RIP3 (1:5000, abcam), p‐RIP3 (1:200, Cell Signaling), MLKL (1:5000, abcam), p‐MLKL (1:5000, abcam), eIF4E (1:5, 000, abcam), p‐eIF4E (1:5, 000, abcam), 4EBP1 (1:5000, Cell Signaling), p‐4EBP1 (1:5000, Cell Signaling), S6K (1:1000, Beyotime), p‐S6K (1:1000, Beyotime), RSK (1:1000, abcam), p‐RSK (1:1000, abcam) and β‐actin (1:5000, Beyotime), and horseradish peroxidase (HRP)‐labeled secondary antibodies (1:5000, Beyotime) individually. Finally, the immunoblots were observed by a chemiluminescence apparatus (Bio‐Rad) and calculated by Image J.

### 5.8 RNA‐Seq

Total RNA was extracted by Trizol (Invitrogen). High‐throughput sequencing was performed by BGI. The differentially expressed genes were analyzed and plotted with heat map and volcano plot in R software.

### 5.9 qPCR assay

qPCR assay was performed by using the SYBR Green PCR kit (Transgene). Data were analyzed by 2^−ΔΔCt^ method. The gene expression was normalized to β‐actin. The specific mRNA primers are as follows: *eIF4E*: Forward 5′‐CTTCTGGCTAGAGACACTGCT‐3, Reverse 5′‐TCTGCGTGGGACTGATAACC‐3′; *4EBP1*: Forward 5′‐ACTCACCTGTGGCCAAAACA‐3, Reverse 5′‐ATTGTGACTCTTCACCGCCT‐3′; *Ccl20*: Forward 5′‐GGCAGAAGCAAGCAACTACG‐3, Reverse 5′‐CTTTGGATCAGCGCACACAG‐3′; *Csf1*: Forward 5′‐AATGCTAACGCCACCGAGAG‐3, Reverse 5′‐TGGAAAGTTCGGACACAGGC‐3′; *β‐actin*: Forward 5′‐TCCTATGTGGGTGACGAGGC‐3, Reverse 5′‐TACATGGCTGGGGTGTTGAA‐3′.

### 5.10 Immunofluorescence staining

HT22 cells were fixed with PF. Cells or tissue sections were blocked with bovine serum albumin, incubated with primary antibodies: RIP3 (1:200), p‐RIP3 (1:100), MLKL (1:200), p‐MLKL (1:200), eIF4E (1:200), p‐eIF4E (1:200,), 4EBP1 (1:200) and p‐4EBP1(1:200), and Alexa conjugated secondary antibodies (1:500, Jackson Immuno Research). Finally, cell or tissue sections were covered by mounting medium and imaged by ZEISS ApoTome or KEYENCE BZ‐X microscope at the same settings.

### 5.11 Transfection approach

The full length of 4EBP1 and mutated hyperphosphorylated 4EBP1 (4EBP1‐2xAsp) were cloned into pcDNA3.1‐3xFlag‐C vector provided by Fenghui Biotechnology. Mutated hypophosphorylated 4EBP1 (4EBP1‐4xAla) was a gift from David Sabatini (Addgene plasmid # 38240). eIF4E, RIP3, and MLKL siRNAs were constructed by Genechem. Transfection procedures were conducted as the instructions. Briefly, plasmids/siRNAs and Lipofectamine 3000/RNAiMax Lipofectamine (Invitrogen) were diluted in the opti‐minimum essential medium (MEM), respectively, and then mixed together. Finally, the mixtures were added to the cultures for transfection.

### 5.12 Co‐IP

Cells were lysed with nondenaturing RIPA lysis containing protease and phosphatase inhibitors. Then, the extracted total protein was incubated with ubiquitin antibody (1:100, Proteintech) coupled with protein A/G agarose beads. Finally, the complex was analyzed by western blotting.

### 5.13 PLA

The in situ interactions of eIF4E with eIF4G or 4EBP1 were detected and visualized by Duolink PLA kit (Merk). The PLA assay was performed as the instructions. Briefly, cell cultures were blocked and incubated with eIF4E and eIF4G (or 4EBP1) antibodies. Then the cultures were incubated with two PLA probes, ligation buffer and amplification buffer. Last, cultures covered by mounting medium and imaged by a fluorescence microscope at the same settings. The eIF4E–eIF4G or eIF4E–4EBP1 dots were quantified by Image J.

### 5.14 Rescue assay

HT22 cells were first transduced with RIP3 or MLKL siRNA. After 4 h, the cells were transduced with 4EBP1 siRNA for another 4 h. Then, cells were cultured for 36 h and injured with TSZ.

### 5.15 Statistical analysis

The results were analyzed using SPSS (Aspire Software International). Means ± SE were calculated from at least three independent experiments and compared by analysis of variance. All statistical tests were two‐sided, and *p *< 0.05 was deemed statistically significant.

## AUTHOR CONTRIBUTIONS

Shuchao Wang, Kun Xiong, Hualin Huang, Bing Jiang, and Jufang Huang designed the study, revised the manuscript. Shuchao Wang and Yun Zhang conducted the experiments, analyzed the data, and prepared the manuscript and images. Meijuan Wang, Zhihao Zhai, Yating Tan, Weiye Xu, Xiaozhen Ren, Ximin Hu, Jinyou Mo, Jia Liu, Yunfeng Yang, and Dan Chen helped to conduct the experiments, analyzed the data and revised the manuscript. All the authors read and approved the final version of the manuscript.

## CONFLICT OF INTEREST STATEMENT

The authors declare no conflicts of interest.

## ETHICS STATEMENT

All animal experiments were approved by the animal research committee of The Second Xiangya Hospital of Central South University in accordance with the US National Institutes of Health (NIH) Guidelines for the Care and Use of Laboratory Animals. APPROVAL NUMBER: 2021136.

## Supporting information



Supporting Information

## Data Availability

All data included in this study are available upon request by contact with the corresponding author.
