# Supplementary material for: Noncanonical feedback loop between “RIP3–MLKL” and “4EBP1–eIF4E” promotes neuronal necroptosis
Source: MedComm (2020). 2025 Feb 18;6(3):e70107. doi: 10.1002/mco2.70107 (PMC11836343; doi:10.1002/mco2.70107)
Supplement: Supplementary file 1 — Supporting Information [file MCO2-6-e70107-s001.docx]

**Non-canonical feedback loop between “RIP3-MLKL” and “4EBP1-eIF4E” promotes neuronal necroptosis**

Shuchao Wang^1, 2, 11, 12, #, *^, Yun Zhang^3, 4, 11, 12, #^, Meijuan Wang^5^, Zhihao Zhai^6^, Yating Tan^2,3^, Weiye Xu^3^, Xiaozhen Ren^3^, Ximin Hu^3^, Jinyou Mo^2^, Jia Liu^2^, Yunfeng Yang^6^, Dan Chen^3, 7^, Bing Jiang^1, 8, 10, 11, *^, Hualin Huang^9, 11, 12, *^, Jufang Huang^3, 7, 10, 11, 12, *^, Kun Xiong^3, 7, *^

^1^ Department of Ophthalmology, The Second Xiangya Hospital of Central South University, Changsha, Hunan, 410011, China.

^2^ Center for Medical Research, The Second Xiangya Hospital of Central South University, Changsha, Hunan, 410011, China.

^3^ Department of Anatomy and Neurobiology, School of Basic Medical Sciences, Central South University, Changsha, Hunan, 410013, China.

^4^ Department of Anesthesiology, The Second Xiangya Hospital of Central South University, Changsha, Hunan, 410011, China.

^5^ Medical Imaging Center, Qingdao West Coast New District People's Hospital, Qingdao, Shandong, 266100, China.

^6^ Department of Neurosurgery, The Eighth Affiliated Hospital, Sun Yat-Sen University, Futian, Shenzhen, 518000, China.

^7^ Hunan Key Laboratory of Ophthalmology, Changsha, Hunan, 410011, China.

^8^ Hunan Clinical Research Center of Ophthalmic Disease, Changsha, Hunan, 410011, China.

^9^ Reproductive Medicine Center, Department of Obstetrics and Gynecology, The Second Xiangya Hospital of Central South University, Changsha, Hunan, 410011, China.

^10^ Department of Radiology, The Second Xiangya Hospital of Central South University, Changsha, Hunan, 410011, China.

^11^ National Clinical Research Center for Mental Disorders, The Second Xiangya Hospital of Central South University, Changsha, Hunan, 410011, China.

^12^ National Center for Mental Disorders, The Second Xiangya Hospital of Central South University, Changsha, Hunan, 410011, China.

^#^ These authors have contributed equally to this work

^*^ Correspondence:

Jufang Huang: huangjufang@csu.edu.cn

Shuchao Wang: wangshuchao@csu.edu.cn

Hualin Huang: huanghualin@csu.edu.cn

Kun Xiong: xiongkun2001@163.com

Bing Jiang: drjiangb@csu.edu.cn


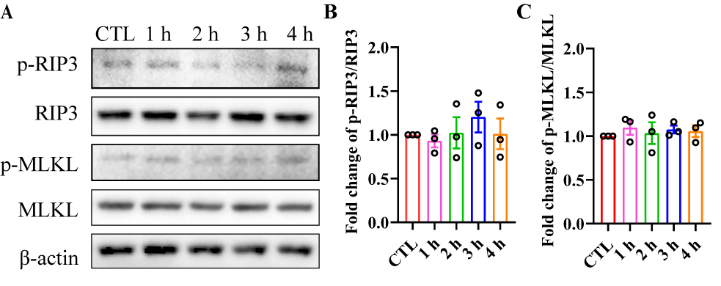


**Figure S1.** (A) Western blot showing p-RIP3/RIP3 and p-MLKL/MLKL expressions in HT22 cells following TS treatment. (B-C) Statistical analysis results of p-4EBP1/4EBP1, p-eIF4E/eIF4E, RIP3 and MLKL expressions.


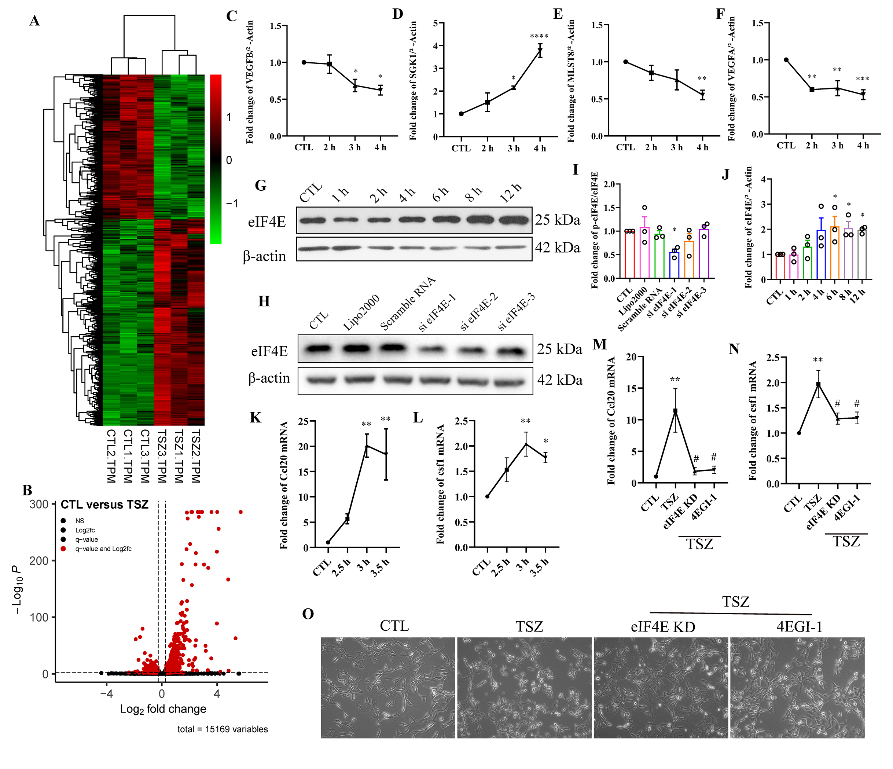


**Figure S2.** (A) Heat map of RNA-seq of HT22 cells after TSZ treatment. (B) Volcano plot showing differentially expressed mRNAs. (C-F, K-N) qPCR results screened the underlying necroptotic genes and showed the Ccl20 and Csf1 productions in HT22 cells following TSZ treatment and pretreatment with eIF4E siRNA and inhibitor before TSZ treatment. (G, H) Western blot showed the eIF4E expression in HT22 cells following a low dosage of TSZ treatment. eIF4E expression after siRNA knockdown. (I, J) Statistical analysis results of eIF4E expression. (O) Phase-contrast images showed the morphological changes in necroptotic cells following TSZ treatment and pretreatment with eIF4E siRNA and inhibitor before TSZ treatment.


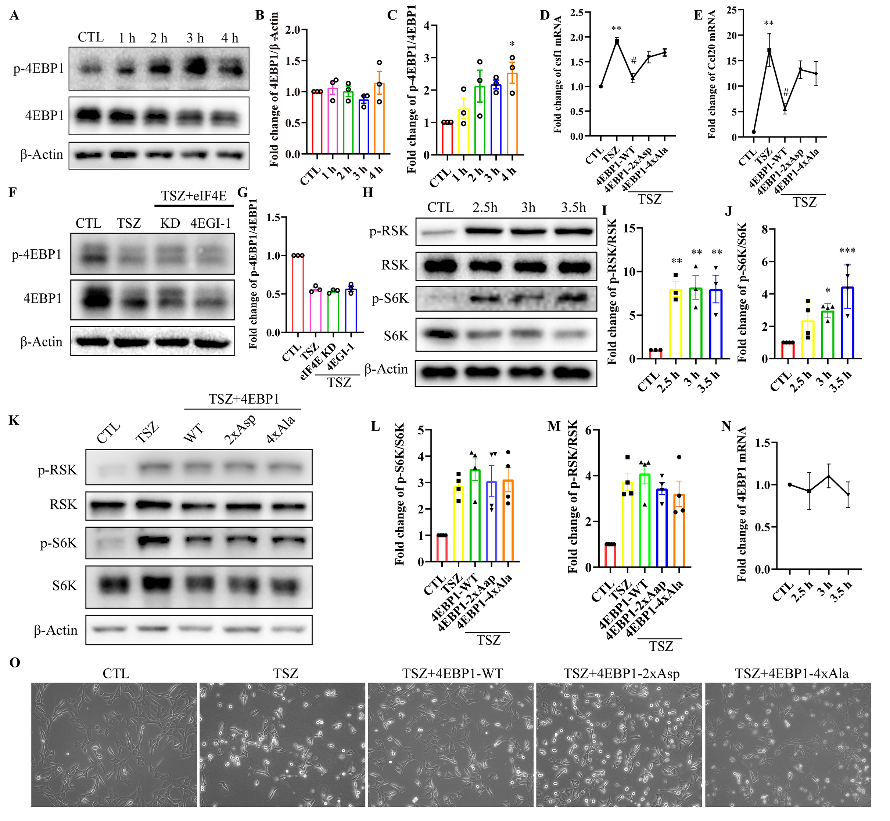


**Figure S3.** (A, F, H, K) Western blot showing p-4EBP1/4EBP1 expression in HT22 cells following TS treatment and pretreatment with eIF4E siRNA and inhibitor before TSZ treatment. p-RSK/RSK and p-S6K/S6K expression following TSZ treatment and pretreated with 4EBP1, 4EBP1-4xAla, and 4EBP1-2xAsp over-expression before TSZ treatment. (B, C, G, I, J, L, M) Statistical analysis results of p-4EBP1/4EBP1, p-RSK/RSK, and p-S6K/S6K expression. (D, E, N) qPCR results showing Ccl20 and Csf1 expressions in HT22 cells following TSZ treatment and pretreatment with 4EBP1, 4EBP1-4xAla and 4EBP1-2xAsp over-expression before TSZ treatment. Relative levels of 4EBP1 mRNA following TSZ treatment. (O) Phase-contrast images showed the morphological changes in necroptotic cells following TSZ treatment and pretreated with 4EBP1, 4EBP1-4xAla, and 4EBP1-2xAsp over-expression before TSZ treatment.





**Figure S4.** (A, B) qPCR results showing the Ccl20 and Csf1 expression in HT22 cells following TSZ treatment and pretreatment with RIP3 and MLKL siRNAs and inhibitors before TSZ treatment.
